# Morphological and molecular identification of *Schizophyllum commune* causing storage bulb rot disease of Lanzhou edible lily in China and its biological characteristics

**DOI:** 10.3389/fmicb.2024.1489999

**Published:** 2024-12-03

**Authors:** Qiaolan Liang, Liexin Wei, Ying’e Chen, Bingliang Xu, Na Zhang

**Affiliations:** College of Plant Protection, Biocontrol Engineering Laboratory of Crop Diseases and Pests of Gansu Province, Gansu Agricultural University, Lanzhou, China

**Keywords:** edible lily bulb rot disease, *Schizophyllum commune*, characterization, molecular identification of a pathogen, Lanzhou edible lily

## Abstract

Lily bulb rot disease has harmed edible lily in recent years, resulting in yield losses in China. As a results, both morphological and molecular techniques must be used to confirm the etiology of storage bulb rot disease on lily bulbs. Lily bulbs with indications of rot symptoms during storage were gathered in Lanzhou, Gansu Province, China. A strain was isolated and its morphologically characterized as a *Schizophyllum commune* specie. Pathogenicity tests further confirmed that the strain caused apparent *S. commune* symptoms on lily bulbs which were consistent with those seen in the field. The pathogenicity of *S. commune* to lily bulb was 100%, and morphological identification showed that the mycelia of the pathogen were white and villous, with septate, branched, and acicular bodies and obvious lock-shaped joints. The mycelia had uneven thickness, ranging from 1.03 to 3.06 μm, and turned gray-white at the later growth stage. Moreover, the pathogen formed a mycelial column on potato dextrose agar (PDA) medium, which formed a coral-like fruiting body primordium, which split, forming pileus for spore production. The spores were colorless and cylindrical, with an oblique tip and a size of 4.6–7.2 μm × 2.2–2.7 μm. The isolate was deduced as based on phylogenetic analysis with 2 genes (ribosomal DNA-ITS and LSU) as well as morphological characteristics and cultural features, the isolate was identified *S. commune*. Soluble starch, yeast extract, temperature of 30°C, pH 7, relative humidity of 100%, and complete dark were shown to be the optimum culture conditions for surface mycelium growth. In conclusion, this is the first report of *S. commune* causing bulb rot of edible lilies in China. The study provides a basis for more effective control strategies for lily bulb rot disease.

## 1 Introduction

Lanzhou lily (*Lilium davidii* var. duch), belonging to the genus *Lilium* of the *Liliaceae* family, is a perennial herbaceous plant with extremely high nutritional value (Dai et al., 2022). Storage diseases of lily have recently surged, resulting in 30–60% loss (Liang, 2011). During storage, the outer layer of the infected bulb forms brown spots with necrotic centers, which gradually spread to the surrounding tissues, resulting in the drying and rotting of the entire bulb. A field investigation found that the disease incidence of lily bulbs was 23.3%, with the disease index of 12.7; however, after storing the lily bulbs in the refrigerator at 2°C for 30 days, the disease incidence reached 100%, and the disease index was 67.0, greatly reducing the quality and commodity value of lily (Gong et al., 2015).

At least 10 species of fungal pathogens are known to cause lily diseases worldwide, *Colletotrichum liliacearum*, *C. lilii*, *C. spaethianum* (Shang et al., 2016; Zhou et al., 2018; Liao et al., 2023), *Botrytis cinerea*, *B. elliptica* (Wu et al., 2015), *Fusarium graminearum*, *F. solani*, *F. tricinctum*, *F. commune*, *F. proliferatum*, *F. moniliforme*, *F. tabacinum*, *F. oxyspora*, and *F. fujikuroi* (Shang et al., 2014; Li et al., 2013; Fang et al., 2021; Song et al., 2022; Liao et al., 2023; Liu et al., 2024; Sun et al., 2024). Viral pathogens such as *Lily mottle virus* and *Lily symptomless virus* (Asjes, 2000) have also been reported to cause lily diseases. Though a common wood rot pathogen, *Schizophyllum* species is rarely reported on lily bulb rot. *Schizophyllum* species, a fungal genus with a wide host range and significant economic relevance as a plant pathogen, comprises a large number of widely distributed morphologically and phylogenetically distinct fungal species (Song et al., 2022). Traditionally, *Schizophyllum* species were identified based on colony morphology, fruiting body characteristics, and other phenotypic characteristics such as the size and shape of macro-and microconidia (Raudaskoski et al., 2012). However, it is difficult to distinguish *Schizophyllum* species based solely on the traditional morphological criteria. Molecular-based techniques have proven particularly effective for identifying and discriminating closely related *Schizophyllum* species (O'Donnell and Cigelnik, 1997). Multi loci phylogenetic analyses of *Schizophyllum* based on the internal transcribed spacer (ITS) and arger subunit ribosormal unit (LSU) have been used to identify the species (Grassi et al., 2018). Identifying the main pathogens causing bulb rot of Lanzhou lily is important for the effective control of storage rot disease and improving the commercial value of edible lily bulbs.

Studies on etiology are essential for a good disease diagnosis and for developing future research on the epidemiology and control of diseases. Therefore, this study used a polyphasic strategy that incorporated morphological features and phylogenetic analysis to identify pathogens causing storage rot disease of edible lily bulbs in China.

## 2 Materials and methods

### 2.1 Plant material

The incidence of lily bulbs in Lanzhou lily planting base (Qilihe District, Lanzhou, Gansu Province, China) was investigated by random method. A total of 500 lily bulbs were investigated, and the investigation was continued after being stored at 2°C for 30 days. The pathogenic fungi were isolated, purified and identified from 60 diseased lily scales with different symptoms.

### 2.2 Isolation and purification of the pathogen

The scales of lily bulbs with disease spots were rinsed with tap water and cut into smaller sections (1 mm × 1 mm) at the junction site of diseased and healthy tissues. The sections were sterilized with 75% alcohol for 1 min, followed by 0.1% mercury for 30 s, and rinsed with sterile water 3–5 times. Thereafter, the sections were dry-blotted using sterilized filter paper and inoculated on potato dextrose agar (PDA) plates (3 pieces per plate). The plates were incubated upside down at 25°C. After 3 days, petri plates with colonies were selected, and a small piece of hyphae was collected from the edge of the colonies and inoculated at the center of new PDA plates for incubation at 25°C. After the spore formation, the pathogen was purified using a single spore isolation method and inoculated into the prepared test tube containing the PDA slant. The formed hyphae were then stored at 4°C for later use.

### 2.3 Pathogenicity determination

The pathogenicity tests were performed on healthy lily scales. Healthy lily scales were cleaned with running tap water for 60 s, surface disinfected with 70% ethanol for 30 s, washed 3 times with sterile distilled water, the isolate strain hyphae and pileus were inoculated on those lily scales to confirm pathogenicity, an empty agar block was placed on lily scales used as a control. Those treatment experiment were repeated three times, a total of 36 lily scales were treated. All treated lily scales were placed in petri dish (wet sponge on the bottom, a piece of sterile filter paper on the top and its middle put a sterile soaked cotton) and kept in an artificial climate chamber (25 ± 1°C and 65% RH). The fungus was re-isolated from the site of disease using the procedures described above and the morphological characteristics were observed under the microscope (Carl Zeiss, China), compared with those of the inoculated isolate to determine the pathogenicity of the isolate strain by Koch’s postulates. Their images were captured via photomicrography using a digital camera, everyday symptoms were observed and documented (SONY DSC-T20, Japan).

### 2.4 Identification of the pathogen

The pathogen was identified by morphological identification and molecular analysis methods. The morphological methods involved observing the growth characteristics, colony color, hypha morphology, spore morphology, and spore size and the molecular analysis methods involved PCR analysis and sequencing. The genomic DNA of the isolate was extracted using the fungal genomic DNA extraction kit (Omega, Shanghai, China). DNA samples were stored at-20°C until they were analyzed. *ITS1/ITS4* primers and *LROR/LR7* primers were used amplify the internal transcribed spacer (ITS), and large subunit ribosomal unit (LSU) genes (Grassi et al., 2018). A 25 μL volume containing 1 μL of DNA template, 1 μL of each primer, 12.5 μL of 2 × Easy TaqPCR Super Mix, and 9.5 μL of ddH_2_O was used for polymerase chain reaction (PCR; Qingke Biotech). Using PCR reactions in a thermal cycler (Model A37028; China). PCR amplification products were visualized on agarose gels and sequenced by Wuhan Jinkailui Bioengineering Co., LTD. (Wuhan, China).

The sequences were amplified from the DNA using the generic fungal primers *ITS-1* (5′-TCCGTAGGTG AACCTGCGG-3′), *ITS-4* (5′-TCCTCCGCTTATTGATATGC-3′), *LSU-LROR* (5′-ACCCGCTGAACTTAAG C-3′), and *LSU-LR7* (5′-TACTACCACCAAGATCT-3′). The ITS and LSU sequences of the strains isolated in this study were subjected to a nucleotide BLAST search using the National Center for Biotechnology Information Database (NCBI: http://www.ncbi.nlm.nih.gov) to obtain the preliminary identifications. GenBank database^1^ was used to compile data sets for phylogenetic analysis. Comparison by used MAFFT 7.4 software, clipping by used Bio Edit 7.0 software, correction and format conversion by used Mesquite 3.5 software. Finally, MEGA 7.0 was used to compare gene sequences to representative sequences from closely related *Schizophyllum* species, and a phylogenetic tree was generated using the neighbor-joining method with 1,000 bootstrap replicates.

### 2.5 Effect of different carbon and nitrogen sources on the growth of the pathogen

The glucose 15.0 g, ammonium sulphate 2.1 g, agar 18.0 g added to 1,000 mL of distilled water as basal medium, lactose 13.5 g, fructose 15.0 g, soluble starch 13.1 g and xylose 13.5 g were used instead of glucose in the basal medium to formulate different carbon source media, respectively. After sterilization, all kinds of media are poured into sterilized petri dishes to make media plates and set aside. On the ultra-clean workbench, the fungus cake was made from the edge of *S. commune* colony cultured for 4 days with a sterilized punch with a diameter of 5 mm, and then inoculated into the above culture medium, respectively. Each treatment was repeated for three times, marked and placed in a constant temperature incubator at 25°C. After 4 days, the growth of the colony was observed, and the diameter of the colony was measured by the “cross” method. To determine the effects of different carbon sources on the growth of *S. commune*.

On the basis of the best carbon source culture medium selected above, ammonium sulfate was replaced by ammonium nitrate 2.11 g, potassium nitrate 4.52 g, urea 0.96 g, peptone 2.8 g and yeast extract 4.7 g, respectively, and made into different nitrogen source culture media. Sterilization, inoculation, culture and “cross” method were carried out in the same way as carbon source determination, so as to determine the influence of different nitrogen sources on the growth of *S. commune*.

### 2.6 Effect of different temperatures, pH levels, relative humidity, and light conditions on the growth of the pathogen

The best carbon and nitrogen source culture medium screened from the experiment in section 2.5 was utilized to determine the radial mycelial growth of the causative pathogen under different temperatures, pH, relative humidity, and light conditions.

Briefly, 45 mL of the sterilized media was poured into three petri plates and inoculated with uniform culture fungus cake (5 mm) from actively growing fungal cultures. The plates were incubated for 4 days under 6 different temperatures (17, 20, 25, 27, 30, and 35°C). After 4 days, the growth of the colony was observed, and the diameter of the colony was measured by the “cross” method. To determine the effects of different temperatures on the growth of *S. commune.*

Adjust the pH of the sterilized culture medium with 0.1 N sodium hydroxide (NaOH) or 0.1 N hydrochloric acid (HCl) to 6, 7, 8, 9 and 10 respectively, 45 mL of the different pH media was poured into three petri plates and inoculated with uniform culture fungus cake (5 mm) from actively growing fungal cultures. The plates were incubated for 4 days under the optimum temperature conditions of the above screened. After 4 days, the growth of the colony was observed, and the diameter of the colony was measured by the “cross” method. To determine the effects of different the value of pH on the growth of *S. commune*.

On the basis of the optimum temperature and pH selected above, the plates were incubated for 4 days under 6 different relative humidity (50, 60, 70, 80, 90, and 100%). After 4 days, the growth of the colony was observed, and the diameter of the colony was measured by the “cross” method. To determine the effects of different relative humidity on the growth of *S. commune*. Besides, on the basis of the optimum temperature, pH and relative humidity selected, the plates were incubated for 4 days under 3 different light conditions (complete light, complete dark and alternate light and dark). After 4 days, the growth of the colony was observed, and the diameter of the colony was measured by the “cross” method. To determine the effects of different light conditions on the growth of *S. commune*.

### 2.7 Data analysis

All statistical analyses were performed using SPSS 21.0. Differences were analyzed using multiple analyses of variance with Duncan’s significant difference tests. A *p* value≤0.05 was considered statistically significant. The data were presented as the mean ± standard error (SE).

## 3 Results

### 3.1 Isolation and pathogenicity analysis of the fungus

**Figure 1:**
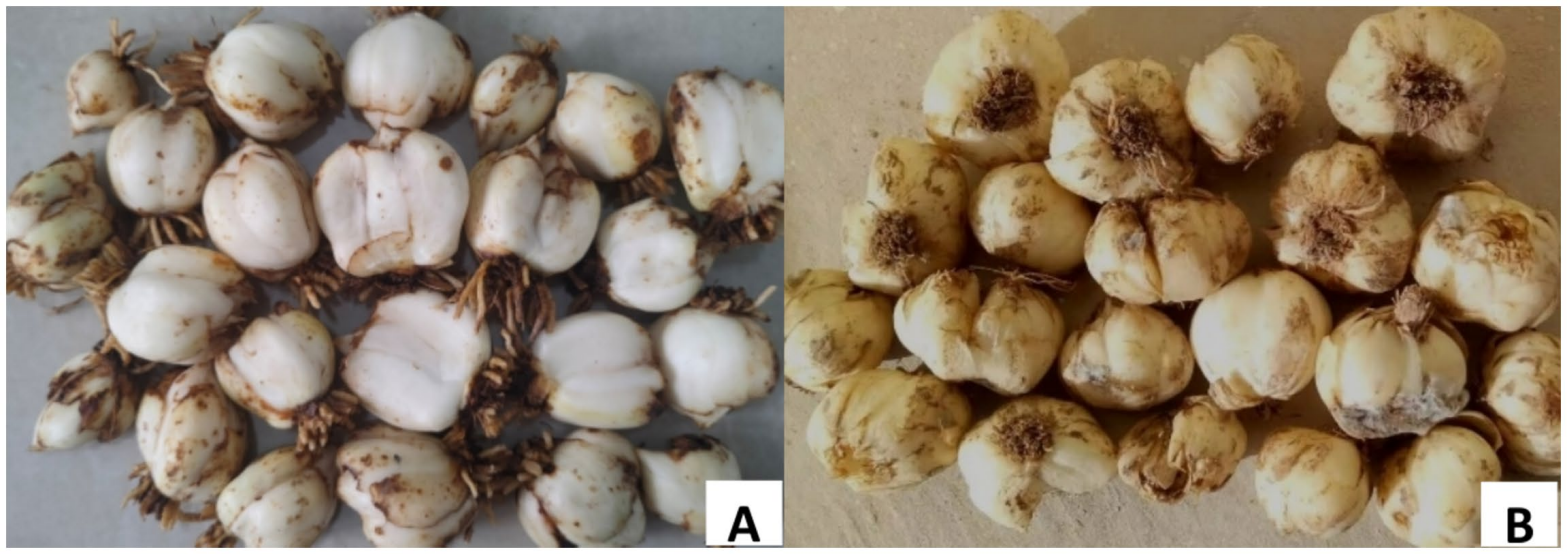
The symptom of Lanzhou lily. **(A)** Before of Lanzhou lily storage. **(B)** After 30 days of Lanzhou lily storage.

**Figure 2:**
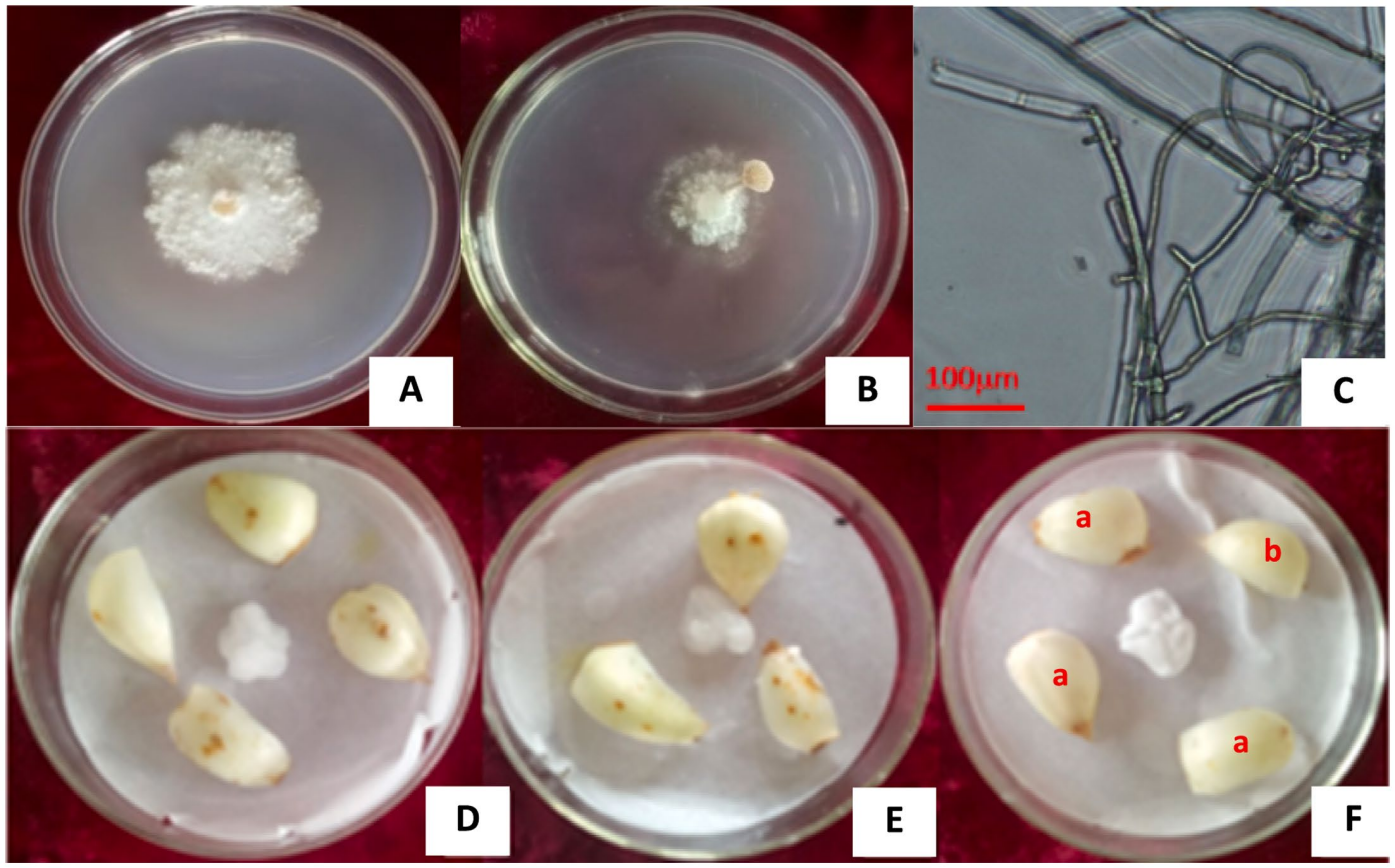
The L1 strain isolated from lily bulbs and its pathogenicity. **(A)** L1 strain pure culture. **(B)** L1 strain fruiting body. **(C)** Microscopic image showing cylindrical and lock-shaped joint mycelia of the L1 strain. **(D,E)** Lily bulbs inoculated with L1 strain hyphae and pileus of fruit body. **(F)** Naturally infected bulb (a) and healthy bulb (b).

Five strains were isolated and purified from 60 diseased lily scales by investigating 500 bulbs stored at 2°C (Figures 1A,B). They are *Fusarium oxysporum*, *Alternaria alternata*, *Botrytis cinerea*, and *Penicillium cyclopium* Westl and an unknown strain, numbered L1 (Figures 2A–C), with isolation rates of 63.33, 33.33, 1.70, 1.70, and 25.00%, respectively. The mycelia and fruiting bodies of isolated L1 strain were inoculated on 12 lily scales for pathogenicity test, respectively. It was found that the scales were yellow-brown disease spots 7 days after inoculation, which were similar to those on natural infected (original) lily bulbs (Figures 2D–F). Both mycelia and fruiting bodies were pathogenic, and the pathogenicity was 100%.

### 3.2 Morphological identification of the fungus causing lily bulb rot disease

Morphological identification showed that the mycelia of the pathogen were white and villous, with septate, branched, and acicular bodies, obvious lock-shaped joints, and uneven thickness ranging from 1.03 to 3.06 μm wide. The mycelia turned gray-white at the later growth stage (Figure 2C).

**Figure 3:**
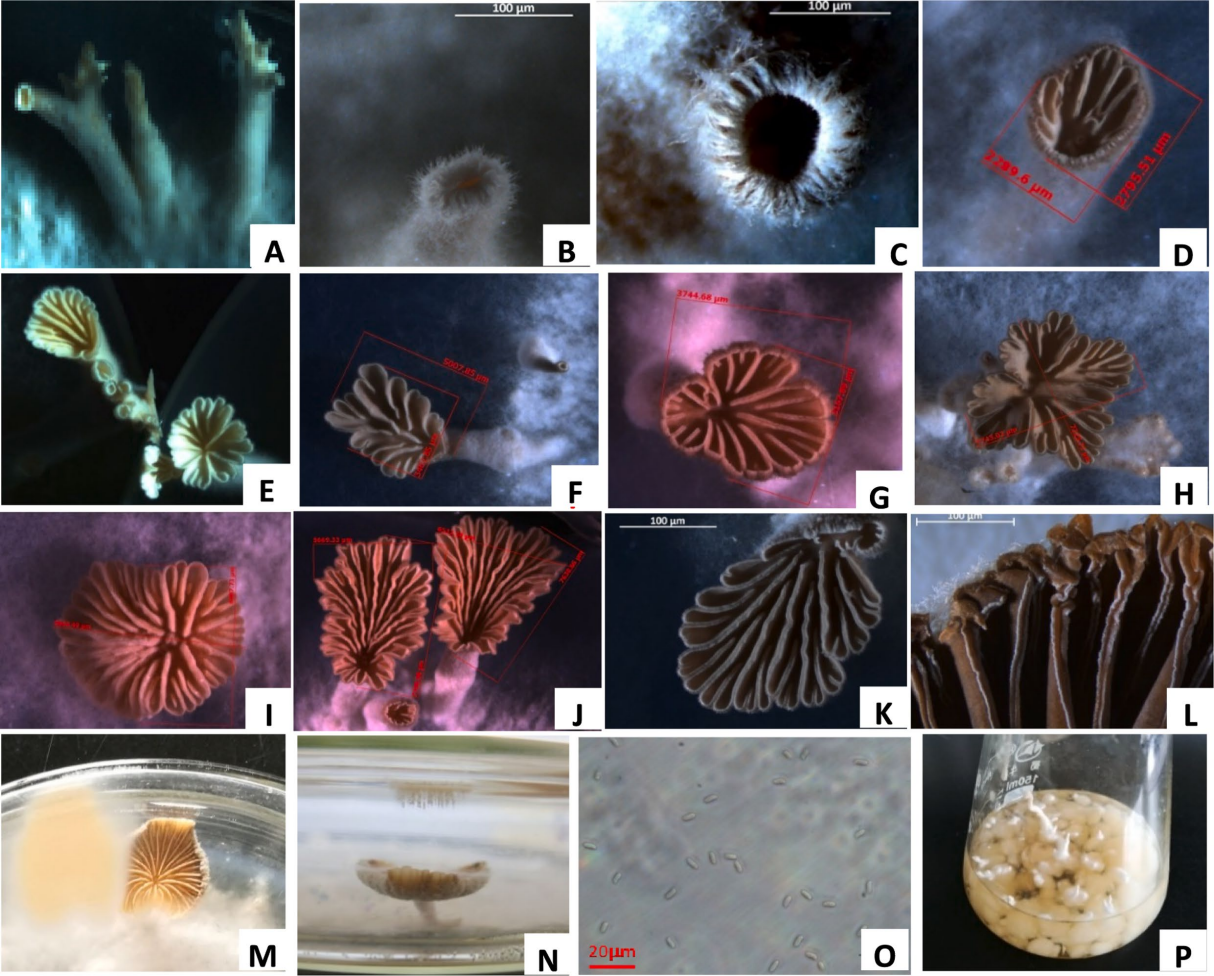
Fruit body formation of *S. commune*. **(A)** Mycelial column of *S. commune* on potato dextrose agar (PDA). **(B)** Coral-like fruiting body primordium (initial stage). **(C)** Split fruiting body primordium. **(D)** pileus of the fruiting body. **(E–K)** Different shapes of fruiting body pileus. **(L)** Gills of the pileus. **(M,N)** Light yellow spore prints. **(O)** Spores ejected from the pileus. **(P)** Mycelial balls and columns produced in the liquid shake flask.

At 25°C, the mycelium column on the PDA medium formed a coral-like fruiting body primordia, which swelled into many sacs that formed a round hole on the top and pod-like split from the side, and these sacs expanded to form fruit bodies (Figures 3A–D). The pileus was pliable, thin-fleshed, white or light yellow, fan or kidney-shaped, with an average diameter of 3.43–10.29 mm. Its surface was densely covered with fluff, and the edges were rolled in and palmately cracked (PDA culture 4 days).

The gills were narrow, linear, non-uniform, white or grey-white, rewinding and radiating from the base with unequal length and longitudinally split along the edge. The stipe was short or absent, and a pile of light yellow spores was formed on the petri dish lid above the pileus (Figures 3E–N). The spores were colorless and cylindrical, with an oblique tip and a size of 4.6–7.2 μm × 2.2–2.7 μm (Figure 3O). The fungal structures were similar to those observed in previous studies (Mao, 2000). After the L1 strain was cultured in a liquid shake flask at 25°C for 4 days, it formed crisscrossed hyphae, which tangled into several small mycelial pellets (Figure 3P). The pellets were white with a sticky texture and different sizes, and their surface had a mycelium column, which formed the fruiting body primordium and fruiting bodies.

### 3.3 Molecular identification of the fungus causing lily bulb rot disease

**Figure 4:**
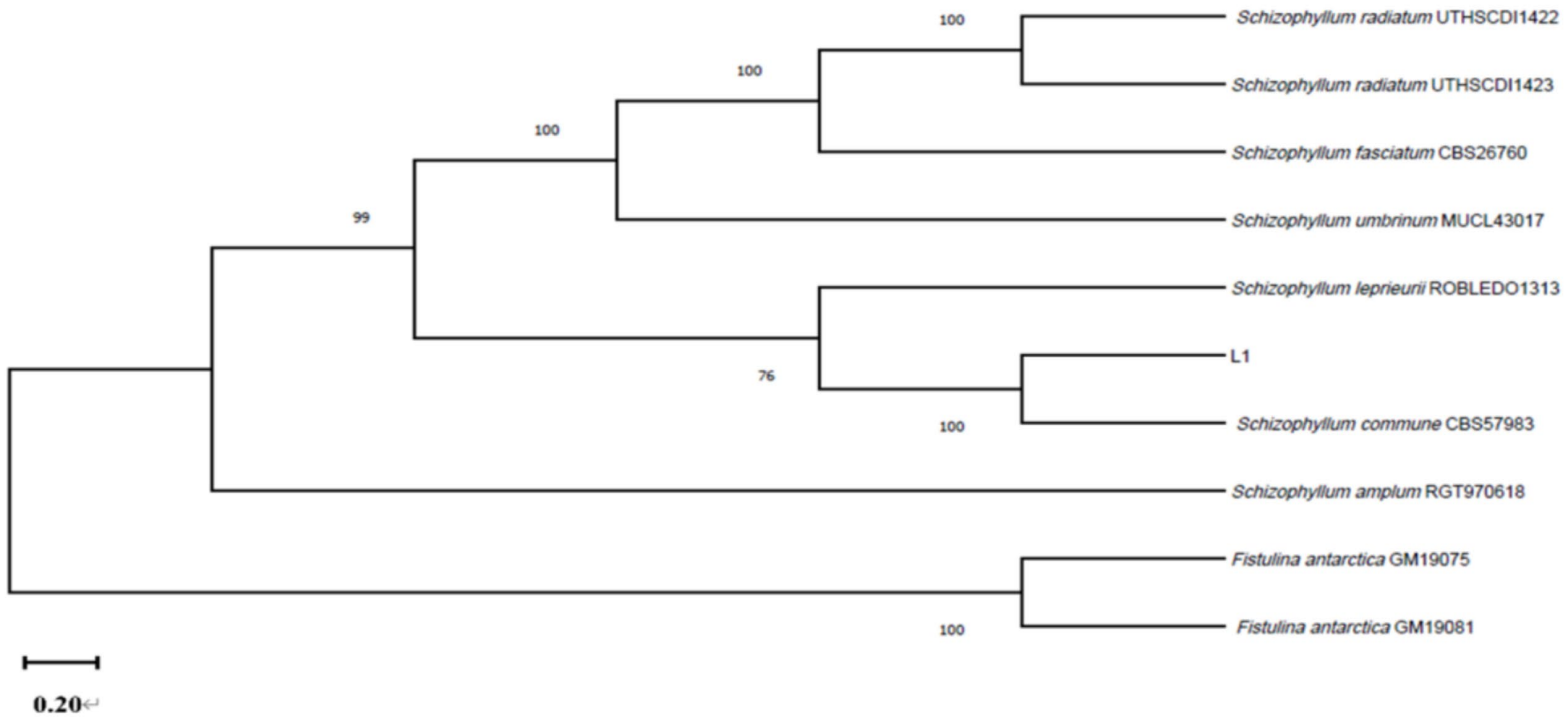
The multilocus phylogenetic tree is constructed by sequences of strain L1 and other sequences.

The DNA of strain L1 was amplified by ITS, and LSU. The 601 bp, and 1,363 bp amplified fragments were obtained by 1% agarose gel electrophoresis, respectively, associated with unique sequence to the strain of *S. commune*. ITS, and LSU sequences were matched to GenBank sequences using blast. ITS, and LSU genes sequences (GenBank Accession Nos: MT251889, and PP082822) all showed 100% identity with *S. commune* CBS57983 (MH861655, MH595605) sequence (Figure 4). Our strain L1 was identified as *S. commune* based on morphological and molecular characteristics.

### 3.4 Effect of different carbon and nitrogen sources on the growth of *S. commune*

**Figure 5:**
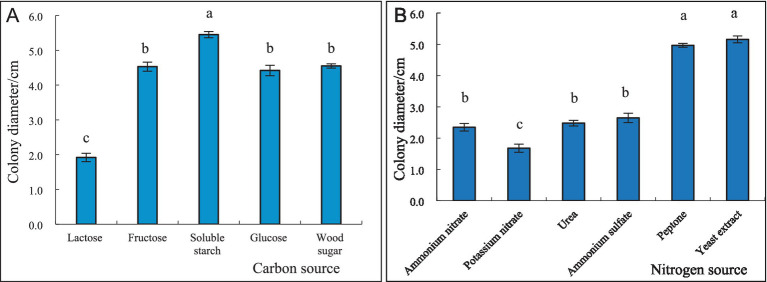
Effect of different carbon and nitrogen sources on the growth of *S. commne*. **(A)** Effect of different carbon sources on the growth of *S. commne*. **(B)** Effect of different nitrogen sources on the growth of *S. commne*.

After 4 days of incubation, the mycelial growth of *S. commune* was the fastest, and its colony diameter was the largest (54.5 mm) when soluble starch was used as the carbon source; however, its growth was slowest, and colony diameter was the smallest (19.2 mm) when lactose was used as carbon source. There was a significant difference between the colony diameters of *S. commune* growing on soluble starch and lactose, and the colony diameters of those growing on other carbon sources were between the two (Figure 5A). Furthermore, the mycelial growth of *S. commune* was the fastest, and its colony diameter was the largest (51.6 mm) when yeast extract was used as the nitrogen source, while the utilization effect of potassium nitrate was the lowest, resulting in the smallest colony diameter (16.8 mm). There was no significant difference in the colony diameter of *S. commune* grown on ammonium nitrate and urea, but there was a significant difference between those growing on other nitrogen sources. The colony diameters of *S. commune* growing on other nitrogen sources were between those grown on yeast extract and potassium nitrate (Figure 5B).

### 3.5 Effect of different temperature, pH levels, relative humidity, and light conditions on the growth of *S. commune*

**Figure 6:**
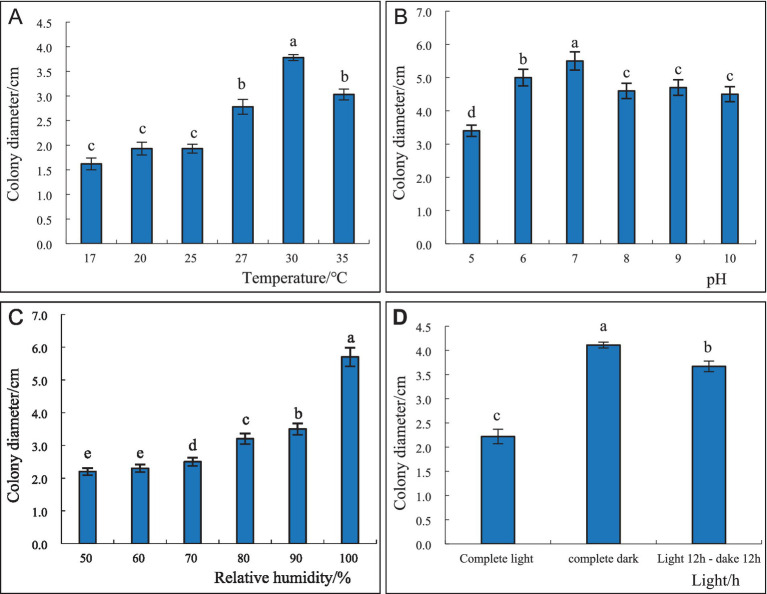
Effect of different temperatures **(A)**, pH levels **(B)**, relative humidity **(C)**, and light conditions **(D)** on the growth and sporulation of *S. commne*.

Soluble starch 15.00 g, yeast extract 4.7 g, agar 18.0 g, 1,000 mL distilled water. The average radial mycelial growth was measured at each temperature after 4 days of incubation, and the results are presented in Figure 6. At 30°C, the highest radial mycelial growth was 37.8 mm, and there were significant differences in colony diameter at different temperatures. Higher temperatures favored the formation of fruiting bodies, with 25°C being the most conducive to the production of the fruiting bodies of *S. commune* (Figure 6A). At pH 7, the maximum radial mycelial growth was 55.0 mm, while minimum radial mycelial growth (34.0 mm) was recorded at pH 5, and the mycelial growth rate at the other pH level was between the two (Figure 6B). The growth of *S. commune* colonies was positively correlated with the relative humidity of the environment; thus, the greater the relative humidity, the better the colony growth. The colony growth of *S. commune* was the best, and the mycelium growth was the densest, with the largest colony diameter (57.0 mm) when the relative humidity reached 100%; however, this humidity level was not conducive to the growth of fruiting bodies. The colony growth was the slowest, with the smallest colony diameter (22.0 mm) when the relative humidity was 50%, but this humidity level was most conducive to the growth of fruiting bodies. There was a significant difference in the colony diameter of *S. commune* growing under 100 and 50% humidity levels, and the colony diameter of those growing under other relative humidity levels were between the two (Figure 6C). Furthermore, continuous darkness of 24 h was the most suitable for the growth of *S. commune*, resulting in the largest colony diameter (41.1 mm). However, the growth of *S. commune* was the slowest under full light conditions, which resulted in the smallest colony diameter (22.2 mm). The colony diameter of *S. commune* under the alternating dark and light conditions was between that under continuous darkness and full light conditions, and there was a significant difference in the diameter of each colony. The full light and alternating dark/light conditions were beneficial to the growth of the fruiting bodies of *S. commune*, while full darkness was beneficial to the growth of mycelium (Figure 6D).

## 4 Discussion

*S. commune* is a facultative parasitic fungus which not only could decay dead and fallen trees, but also could infect living trees, causing white rot in the xylem and eventually causing tree death (Li, 1985; Wang and Huo, 2006; Sun, 2019). *S. commune* infection rate is significantly elevated in the wound and the primary rod, with decay areas (Adaskaveg et al., 1993). The pathogen can cause white wood decay (Mao, 2000), apple tree rot, and peach tree rot, with incidence rates of up to 100%, and the longer the age of the tree, the higher the incidence rate (Bergdahl and French, 1985; Zeng et al., 2008). *S. commune* is drought-tolerant and light-loving with a wide range of mycelial growth temperatures, reproduces rapidly, and contains a highly active cellulase enzyme that reaches deep into the xylem, thus greatly impacting the infested trees (Bergdahl and French, 1985). As a “medicinal and food” crop, Lanzhou lily is rich in carbohydrates, minerals, vitamins, proteins, and dietary fibre and thus has a high nutritional value (Dai, 2005). After 9-year-old lily bulbs are dug from the field, they are mostly subjected to low-temperature or cold storage, and vacuum-packed after peeling off the decayed bulbs before sale; however, when the temperature rises, even the vacuum-packed lily bulbs cause become seriously decayed due to the amplification of the pathogens they carry, therefore affecting their consumption (Shang et al., 2014). It has been found that lily storage-phase diseases are caused by various pathogens, with the most common being *F. oxysporum*, *Rhizopus stolonifer*, and *Penicillium cyclopium*, among others (Liang, 2011; Gong et al., 2015). In this study, the identified fungal pathogen, *S. commune* could also cause bulb rot of lily by wound infestation of Lanzhou lily bulbs, making it the first report of *S. commune* as the causative agent of storage rot disease in Lanzhou lilies in China.

The results showed that *S. commune* could slow growth in an environment below 17°C optimum growth temperature was 30°C, and the optimum relative humidity was 100%, indicating that the storage disease of lily bulbs was more serious at higher temperatures and higher humidity. Moreover, the best growth of the pathogen was at pH 6.0 in total darkness, with starch as the carbon source, and the growth was poorer in the medium containing nitrogen than in the medium containing carbon. The results of this study are somewhat different from a previous study on the decay pathogen, *S. commune* of torrefied trees, which found that the fungus could grow at 15–35°C, with 20–25°C as the optimal growth temperature range, and that *S. commune* grew optimally when the carbon source was fructose (Wang et al., 2009). However, our results were consistent with a study by Liang et al. (2022) which reported that the optimum temperature for the growth of *F. schizothoracinum*, the causal agent of pepper tree rot disease, was 30°C, but the results of the study on the optimum growth relative humidity, pH, and carbon source were somewhat different. There are many different research results on the optimal pH range for the growth of *Schizosaccharomyces pombe*. Chen (1986) reported that the optimal pH range for the growth of *S. pombe* was 5.0–6.0, and its mycelial growth ceased at pH less than 5.0 and more than 10.0. Moreover, Lu and Lu (2011) found that the optimum pH range of the fungus was 4.5–5.5, with a humidity of 70% and a temperature range of 8–34°C, and the optimum growth occurring at the temperature range of 23–26°C. This indicated that different hosts influence the optimum growth conditions of *S. pombe* and that the cause of such differences might be related to the environmental growth conditions of the host and host species.

In summary, *S. commune*. Was found to be the causative pathogen of storage bulb rot of Lanzhou lilies in China. The pathogen was identified through morphological characterisation, molecular characterisation based on ITS and LSU sequences and pathogenicity analysis. The optimal culture conditions of the pathogen were soluble starch for the carbon source, ammonium sulphate for the nitrogen source, 30°C, pH 7, relative humidity of 100%, and total darkness. To our knowledge, this is the first report of *S. commune* as a causative agent of storage rot disease in Lanzhou lilies in China. The results lay the foundation for developing a safe and effective method of disinfecting and treating lily storage cold rooms and for pre-treating lily bulbs to prevent and control bulb rot disease caused by *S. commune*. Thus, the study provides a basis for more effective disease control strategies. However, in this study, only the morphological and biological characteristics of *S. commune* were studied, but the biological characteristics of *F. oxysporum*, *A. alternata*, *B. cinerea*, and *P. cyclopium* Westl and the pathogenicity of these five pathogens alone and combined infection lily bulbs need further research.

## Data Availability

The original contributions presented in the study are included in the article/Supplementary material, further inquiries can be directed to the corresponding author.

## Footnotes

^1^ http://www.ncbi.nlm.nih.gov
